# The Effect of Antioxidant Administration on Semen Quality in Men with Infertility: A Randomized Placebo-Controlled Clinical Trial

**DOI:** 10.3390/antiox14040488

**Published:** 2025-04-18

**Authors:** Pinelopi Ioannidou, Theodosia Zeginiadou, Christos Venetis, Dimitrios Papanikolaou, Leonidas Zepiridis, Despoina Savvaidou, Katerina Chatzimeletiou, Alexandros Lambropoulos, Dimitrios G. Goulis, Grigoris Grimbizis, Efstratios M. Kolibianakis

**Affiliations:** 1 Unit for Human Reproduction, 1st Department of Obstetrics-Gynecology, School of Medicine, Aristotle University of Thessaloniki, Papageorgiou General Hospital, 56403 Thessaloniki, Greece; 2 Andrologylab, Andrology Diagnostic Center, 54624 Thessaloniki, Greece; 3 Centre for Big Data Research in Health, Faculty of Medicine & Health, University of New South Wales, Sydney, NSW 2052, Australia; 4 2nd Department of Urology, School of Medicine, Aristotle University of Thessaloniki, Papageorgiou General Hospital, 54124 Thessaloniki, Greece; papanikd@auth.gr; 5 Laboratory of Medical Biology—Genetics, Faculty of Medicine, School of Health Sciences, Aristotle University of Thessaloniki, 54124 Thessaloniki, Greece; 6 Unit of Reproductive Endocrinology, 1st Department of Obstetrics and Gynecology, Aristotle University of Thessaloniki, 56429 Thessaloniki, Greece

**Keywords:** antioxidants, male infertility, placebo, Spermotrend^®^

## Abstract

A randomized, placebo-controlled, quadruple-blind trial was performed to evaluate the effect of oral administration of the antioxidant combination Spermotrend^®^ for three months on semen quality in infertile men with at least one abnormal variable in semen analysis. Eighty men were randomized between 2019 and 2022, receiving either the antioxidant combination Spermotrend^®^ (*n* = 40, spermotrend-group) or placebo (*n* = 40, placebo-group). Although a total of 80 patients were enrolled in the study, the final data is only from 70 patients. The primary outcome measure was sperm motility (rapid progressive, progressive, and total motility). The values of primary and secondary outcomes between treatment initiation and treatment completion were compared within groups. Moreover, their changes between treatment initiation and treatment completion were compared between the placebo- and the spermotrend-groups. Sperm rapid progressive motility significantly increased in infertile men treated for three months with antioxidant combination Spermotrend^®^ (+1.0%, 95% CI: 0.0 to +2.0, *p* = 0.04), while this increase was not observed in the placebo-group. Sperm progressive motility significantly increased in infertile men treated for three months with antioxidant combination Spermotrend^®^ (+3.0%, 95% CI: 0.0 to +15.1, *p* = 0.02), while this increase was not observed in the placebo-group. Similarly, DFI was significantly decreased in infertile men treated for three months by antioxidant combination Spermotrend^®^ (−3.2%, 95% CI: −5.8 to −0.5, *p* = 0.02). However, no statistically significant differences were observed in the changes of pre- and post-treatment values between the spermotrend- and the placebo-group regarding sperm progressive motility, concentration, normal morphology, DFI, and formation of 8-OH-dG. The antioxidant combination Spermotrend^®^ appears to exert limited benefit on sperm motility and DFI in infertile men with at least one abnormal variable in semen analysis.

## 1. Introduction

Male infertility represents a significant health burden for modern societies as it has been estimated that 40% of couples will be impacted by a form of male factor infertility [1]. Despite the significant advances in the management of female factor infertility over the past few decades, the causes and treatment of male factor infertility have been understudied. One of the emerging fields of interest in the past decade has been the effect of oxidative stress on sperm function and its potential clinical implications in terms of reproductive outcome.

Oxidative stress is defined as an imbalance between the production of reactive oxygen species (ROS) and the body’s ability to neutralize these harmful molecules through antioxidants. This imbalance can lead to cellular damage, particularly in sperm, where oxidative stress negatively affects sperm motility, viability, DNA integrity, and mitochondrial function, all of which are essential for successful fertilization [2]. Elevated levels of oxidative stress have been identified as a major factor contributing to male infertility, impairing sperm function and potentially reducing the chances of conception [3].

According to the World Health Organization (WHO) guidelines (5th edition, 2010), semen analysis parameters such as sperm concentration, motility, and morphology are used to assess semen quality and diagnose abnormalities [4]. For instance, sperm concentration below 15 million/mL, progressive motility below 32%, and a normal morphology rate of less than 4% are indicative of conditions like oligozoospermia, asthenozoospermia, and teratozoospermia. These criteria help classify the severity of male infertility and guide treatment decisions.

Considering the above, several antioxidants have been evaluated, either in isolation or in combination, regarding their potential to improve semen quality and, eventually, the probability of pregnancy [5,6,7,8,9,10].

A recent Cochrane meta-analysis suggested that treatment with combinations of antioxidants as compared to placebo or no treatment in men with infertility is associated with an increase in sperm motility [mean difference (MD) +12.71, 95% confidence interval (CI) +11.33 to +14.08, 7 studies, 684 patients] [11]. However, no improvement was observed in the probability of live birth and DNA fragmentation index (DFI) levels. However, since data synthesis in the above meta-analysis was performed by combining data from studies comparing different combinations of antioxidants, the external validity of the results obtained is decreased, limiting their clinical usefulness.

Currently, the widespread use of a combination of antioxidants is either not supported by relevant randomized controlled trials (RCTs) or, where these trials are present, they are of poor quality, inhibiting informed clinical decision-making [12].

The aim of the study was to evaluate the effect of oral administration of the antioxidant combination Spermotrend^®^ for three months on semen quality in infertile men with at least one abnormal variable in semen analysis.

## 2. Materials and Methods

### 2.1. Study Population

From 2019 to 2022, 80 infertile men were randomized to either Spermotrend^®^ administration (*n* = 40, spermotrend-group) or placebo (*n* = 40, placebo-group) for three consecutive months. Randomization was performed using a randomization list by a physician who was not involved in the outpatient clinic of the Unit. This was a quadruple-blind RCT (participants, clinicians, outcome assessors, data analyst). Patients could participate in the study only once (Trial registration number: NCT04256278).

Patients were recruited in the outpatient clinic at the Unit for Human Reproduction of the 1st Department of Obstetrics and Gynecology at the Aristotle University of Thessaloniki. They were evaluated for semen variables (according to the WHO 2010 criteria to ensure standardization), DFI, and the formation of 8-hydroxy-2-deoxy-guanosine (8-OHdG) by detecting 8-OHdG peroxidation product on sperm at a collaborating andrology diagnostic center (Andrologylab). Pre-treatment evaluation took place following randomization and before treatment initiation with either the antioxidant combination Spermotrend^®^ or placebo, while post-treatment evaluation was performed within a week after completion of treatment. All semen analyses were performed in the same andrology laboratory using identical equipment and protocols throughout the study period. Importantly, all samples were evaluated by the same experienced laboratory technician who was blinded to the group allocation, in order to minimize inter-observer variability. The study was approved by the Ethics Committee Review Board of Aristotle University of Thessaloniki. Written consent was obtained from all patients.

Inclusion criteria: Men with infertility; 18–50 years old; no treatment for infertility in the last three months; normal hormone profile (TSH, FSH, LH, total testosterone, prolactin); normal sperm culture (aerobic, anaerobic bacteria, mycoplasma, ureaplasma, and chlamydia); physiological scrotal ultrasound.

Infertility was defined by the failure to achieve a pregnancy after 12 months or more of regular unprotected sexual intercourse [4]. All patients should have at least one out-of-reference-range variable in semen analysis (concentration, motility, morphology) according to the World Health Organization (WHO) 2010 criteria.

Exclusion criteria: Genetic cause of infertility; history of cryptorchidism, orchiectomy, testicular cancer, severe heart, liver, or kidney disease, or endocrine disease (primary or secondary hypogonadism, hyperprolactinemia, thyroid, and pituitary or adrenal disease); history of systemic disease or treatment in the last three months; body mass index (BMI) > 30 kg/m^2^; participation in another study.

### 2.2. Description of the Intervention and the Measured Parameters

Both the antioxidant combination Spermotrend^®^ and placebo were provided by ARMATURA, Greece, and were administered for three months based on the randomization procedure.

The supplement (Spermotrend^®^, CATALYSIS, S.L., Madrid, Spain) contains the following molecularly activated antioxidants (https://www.catalysis.es) accessed on the 1 September 2024): fructose 104.0 mg, African plum (Pygeum africanum hook) 100.0 mg, L-arginine 50.0 mg, L-carnitine 40 mg, (l-ascorbic acid) 30.0 mg, zinc sulfate 20.0 mg, vitamin e (d-alpha tocopherol) 5.0 mg, vitamin B_6_ (pyridoxine hydrochloride) 1.0 mg, folic acid (pteroilmonoglutamycin b 12 mg acid) 100.0 mg, (cyanocobalamin) 0.5 μg, and bulking agent (microcrystalline cellulose) 100.0 mg.

### 2.3. Outcome Measures

The primary outcome measure was sperm motility (rapid progressive, progressive, and total motility), while secondary outcome measures were sperm concentration, normal morphology, formation of 8-OH-dG, and DFI concentration.

### 2.4. Assessment of Outcome Measures

Sperm motility, concentration, and normal morphology were evaluated according to WHO criteria [13]. DFI was determined by flow cytometry [14], and formation of 8-OH-dG was assessed by detecting 8-OHdG peroxidation product on sperm. 8-OHdG peroxidation product on sperm is the oxidized derivative of guanine, the nucleotide most susceptible to oxidation and is an indicator of DNA oxidation with high specificity [15]. A percentage of sperm with DNA fragmentation > 25% was considered to be of low quality [16].

### 2.5. Sample Size

Sample size estimation, using PASS 11 [17], showed that 36 patients were required in each group, assuming a mean difference of 10% [18] between the changes in motility before and after treatment between the groups compared, and using a standard deviation of 15. Assuming that a proportion of patients (10%) would not complete the study, the sample size was increased to 80 men.

### 2.6. Statistical Analysis

The values of primary and secondary outcomes between treatment initiation and treatment completion were compared within groups. Moreover, their changes between treatment initiation and treatment completion were compared between the placebo- and the spermotrend-groups.

The normality of the distribution of continuous variables was evaluated using the Shapiro–Wilk test. Non-normally distributed continuous variables were described using the median (95% CI of the median), whereas normally distributed variables were described using the mean (95% CI of the mean). Binary variables are described using proportions (95% CI). Wilcoxon-matched pairs signed-rank test was used to compare non-normally distributed continuous variables, whereas the paired t-test was used to compare normally distributed continuous variables. For non-normally distributed variables, the median difference between members of the groups compared was estimated using the Hodges–Lehman median difference. In contrast, for normally distributed variables, the difference was calculated using the *t*-test. A test of equality of medians was performed using Quantile regression [19]. In a post-hoc analysis, primary and secondary outcome measures were, in addition, evaluated in men with DFI greater or lower than 25%. All statistical analyses were performed using STATA (Version 17, Stata Corp LP, College Station, TX, USA). Statistical significance was set at *p* = 0.05.

## 3. Results

**Figure 1:**
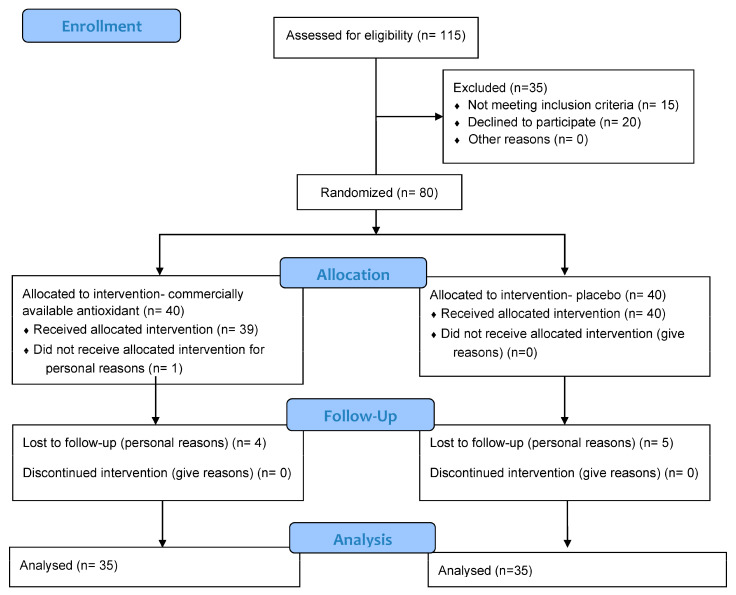
Flow diagram of the study according to the Consort 2010 statement, showing the flow of participants through each stage of a randomized trial.

Eighty men with infertility were randomized to receive either the antioxidant combination Spermotrend^®^ (*n* = 40, spermotrend-group) or placebo (*n* = 40, placebo-group) for three consecutive months. Ten patients dropped out from the study (five in the spermotrend-group and five in the placebo-group). Nine patients did not perform the second semen analysis after the completion of treatment (spermotrend-group: *n* = 4, placebo-group: *n* = 5), while one patient in the spermotrend-group did not receive the allocated intervention. Thus, 35 patients completed the study in the spermotrend-group and 35 patients in the placebo-group (Figure 1).

Baseline characteristics of patients randomized in the spermotrend-group and in the placebo-group are shown in Supplementary Table S1. Male age, BMI (kg/m^2^), smoking, exercise, alcohol consumption, and primary infertility were similar between the two groups.

### 3.1. Pre-Treatment and Post-Treatment Semen Parameters of Patients Who Completed the Study in the Spermotrend- and Placebo-Groups

Pre-treatment and post-treatment semen parameters of patients who completed the study in the spermotrend- and placebo-groups are shown in Table 1.

Changes between pre- and post-treatment values in the placebo- and the spermotrend-groups are shown in Table 2.

Sperm rapid progressive and sperm progressive motility significantly increased in men treated for three months with the antioxidant combination Spermotrend^®^, while this increase was not observed in the placebo-group (Table 2).

DFI was significantly decreased in men treated for three months with the antioxidant combination Spermotrend^®^, while this decrease was not observed in the placebo-group (Table 2).

Formation of 8-OH-dG significantly decreased in men treated for three months either with the antioxidant combination Spermotrend^®^ or placebo (Table 2).

On the other hand, no statistically significant differences were observed in infertile men who were treated for three months with either the antioxidant combination Spermotrend^®^ or placebo between pre- and post-treatment values regarding total sperm motility, sperm concentration, and morphology (Table 2).

No statistically significant differences were observed in the changes of pre- and post-treatment values between the spermotrend- and the placebo-group regarding sperm motility, sperm progressive motility, sperm morphology, sperm concentration, DFI, and formation of 8-OH-dG (Table 3).

A sensitivity analysis was performed on patients depending on the pre-treatment value of DFI using a threshold of 25%.

### 3.2. Infertile Men with DFI > 25%

Sperm rapid progressive motility significantly increased in infertile men with DFI > 25% who were treated for three months with the antioxidant combination Spermotrend^®^, while this increase was not observed in the placebo-group. Similarly, DFI was significantly decreased in men with DFI > 25% who were treated for three months with the antioxidant combination Spermotrend^®^, while this decrease was not observed in the placebo-group (Supplementary Table S2).

Formation of 8-OH-dG significantly decreased in infertile men with DFI > 25% who were treated for three months with placebo, while this decrease was not observed in the spermotrend-group (Supplementary Table S2).

Νo statistically significant differences were observed in infertile men with DFI > 25% who were treated with either Spermotrend^®^ or placebo between pre- and post-treatment values regarding sperm total motility and sperm progressive motility (Supplementary Table S2).

### 3.3. Infertile Men with DFI < 25%

Formation of 8-OH-dG significantly decreased in infertile men with DFI < 25% who were treated for three months with either the antioxidant combination Spermotrend^®^ or placebo (Supplementary Table S2).

Νo statistically significant differences were observed in infertile men with DFI < 25% who were treated with either Spermotrend^®^ or placebo between pre- and post-treatment values regarding sperm total motility, sperm rapid progressive motility, sperm progressive motility, and DFI (Supplementary Table S2).

A statistically significant difference was observed in the changes of pre- and post-treatment values between the spermotrend and the placebo group regarding sperm rapid progressive motility in infertile men with DFI > 25% (Supplementary Table S3).

No statistically significant differences were observed in the changes of pre- and post-treatment values between the spermotrend- and the placebo-group regarding sperm motility, sperm progressive motility, sperm morphology, sperm concentration, DFI, and formation of 8-OH-dG in patients depending on the pre-treatment value of DFI (Supplementary Table S3).

All patients were asked for the occurrence of any adverse effects after completion of treatment with either the antioxidant combination Spermotrend^®^ or placebo. No adverse effects were reported by all patients participating in the study for the duration of follow-up.

## 4. Discussion

The current RCT shows that, in infertile men with at least one abnormal semen variable, the antioxidant combination Spermotrend^®^ as compared to placebo appears to exert limited benefit on sperm progressive motility, sperm rapid progressive motility, and DFI, by comparing pre- and post-treatment values. On the other hand, no significant differences were observed in the changes of pre- and post-treatment values between the spermotrend- and the placebo-groups for all semen parameters examined.

The observed 1% increase in rapid progressive motility is small and its clinical relevance, if it represents a real finding, is probably limited. However, it cannot be excluded that even slight improvements in rapid progressive motility could enhance the chances of successful fertilization, as sperm with rapid forward movement are more likely to reach and penetrate the oocyte [20]. The increase in rapid progressive motility is not accompanied by an increase in overall motility. This might be explained by an improvement in mitochondrial function, energy metabolism, or antioxidant protection, which specifically benefits the most motile sperm subpopulation [21].

Behavioral changes during the treatment period may have influenced the results, independent of the supplement’s direct effects. Previous studies suggest that taking a supplement, regardless of whether it is an active treatment or a placebo, can lead to behavioral modifications [22]. Participants may become more conscious of their reproductive health, leading to lifestyle adjustments that positively affect semen quality. However, since this is an RCT, these behavioral changes are expected to affect both groups. As a result, the effect of spermotrend treatment can be reliably assessed. 

Numerous studies have suggested that even being overweight can adversely affect male fertility by contributing to hormonal imbalances, reduced sperm quality, and increased oxidative stress, which are known to impair sperm motility and DNA integrity [23]. Relevant subgroup analysis could be conducted in future studies specifically designed and powered for this purpose.

The present study is the first RCT to evaluate whether administration of Spermotrend^®^ as compared to a placebo for three months improves sperm quality in men with infertility. Although the current study assessed the benefit of the antioxidant combination Spermotrend^®^ treatment, it was not designed to evaluate its effect on the probability of pregnancy occurring either spontaneously or after Medically Assisted Reproduction (MAR).

The current RCT did not explicitly include men with abnormal DFI concentration, limiting the extrapolation of the results obtained from this group of patients. However, subgroup analysis taking into account the pre-treatment values of DFI showed an improvement in sperm rapid progressive motility and a decrease in DFI when the pre-treatment value of DFI > 25%, which were not observed in the placebo-group.

The current study was designed to detect a 10% difference in sperm motility between the pre- and post-treatment values changes in the spermotrend- and placebo-groups [18]. Thus, it cannot be excluded that administering antioxidant combination Spermotrend^®^ for three months could result in sperm motility improvements of smaller magnitude.

An increase in progressive motility between pre- and post-treatment values was observed in the spermotrend-group, but not in the placebo-group (Table 2). This is in line with RCTs comparing combined antioxidants with placebo [24,25,26]. However, evidence also exist to suggest that antioxidant administration does not improve sperm motility [27,28,29,30,31,32]. In the above studies, however, different antioxidants have been administered in patients with different characteristics compared to those evaluated in the current study.

Moreover, a decrease in sperm DFI was observed between pre- and post-treatment values in the spermotrend-group and not in the placebo-group (Table 2). Similar improvements in sperm DFI following antioxidant administration have been reported [31]; although evidence also exist to suggest that DFI is not improved by antioxidant administration or that it can even deteriorate [32,33].

Our findings diverge from those reported in a placebo-controlled trial, which also investigated the effects of antioxidants on male fertility [34]. However, in our study, the magnitude of the improvements was less pronounced, potentially due to differences in the specific antioxidant agents utilized, the dose, or the treatment duration. These differences highlight the complexity of evaluating antioxidant supplementation on male fertility, underscoring the need for further research to optimize treatment protocols. On the other hand, our findings are consistent with those reported in a placebo-controlled trial, indicating that antioxidants do not lead to significant improvements in semen parameters [33].

Furthermore, additional research is needed to fully understand the long-term effects and potential side effects of antioxidant supplementation on male fertility. Future trials should aim to standardize treatment protocols and assess the clinical relevance of small improvements in sperm quality, particularly in relation to pregnancy and live birth rates.

Theoretically, the lack of effect of Spermotrend^®^ on DFI and motility might be due to the relatively low dose of many components in the antioxidant combination evaluated. The spermotrend contains only 30 mg of Vitamin C and 5 mg of Vitamin E, both being considered very low doses of these antioxidants. Previous trials have used doses of Vitamin C at around 500–1000 mg and Vitamin E at typically 300–600 mg/day [5,35]. Similarly, the 20 mg dose of zinc used in this RCT is low compared to the dose used in previous studies ranging from 66mg to 400 mg [36,37]. On the other hand, the modest improvement in sperm motility might not be associated with the antioxidant action of Spermotrend^®^ but instead might be due to the presence of fructose in the antioxidant preparation evaluated. Fructose is the main carbohydrate found in seminal plasma, where it can act as an energy source for sperm motility independently of antioxidant action. 

Recently, the role of prostasomes in male infertility was evaluated, particularly their involvement in protecting sperm from oxidative stress. Prostasomes, secreted by the prostate, contain various enzymes and antioxidants that help mitigate oxidative damage to spermatozoa. These vesicles play a vital role in maintaining sperm motility and protecting sperm DNA, which are critical for successful fertilization. The presence and functional integrity of prostasomes may be a key factor in modulating sperm quality [38]. Additionally, future studies should investigate the extent to which antioxidant therapy, specifically spermotrend, affects the presence and functionality of prostasomes, as well as the mechanism through which it ultimately influences sperm motility.

Recent advancements in oxidative stress-related mechanisms have identified cysteine trioxidation as a novel biomarker of oxidative damage. Cysteine trioxidation reflects the degree of oxidative modification of cysteine residues in proteins, offering a sensitive measure of oxidative stress in cells. This biomarker may provide deeper insights into the mechanisms underlying male infertility and the potential efficacy of antioxidant therapies. Given that oxidative stress plays a pivotal role in sperm dysfunction, the use of cysteine trioxidation as a biomarker could enhance our understanding of how antioxidants modulate oxidative damage and improve sperm quality [39,40]. Future studies should explore the use of cysteine trioxidation in clinical settings to assess the effectiveness of antioxidant treatments for male infertility.

Currently, many questions remain unanswered regarding the use of antioxidant preparations in clinical practice. For instance, the optimal duration of antioxidant administration or the optimal doses of antioxidants have yet to be determined. Similarly, antioxidant treatment has not been evaluated in men with oxidative stress or high DFI, as assessed by relevant tests prior to initiation of treatment [1].

## 5. Conclusions

The antioxidant combination Spermotrend^®^ appears to exert limited benefit on sperm motility and DFI in infertile men with at least one abnormal variable in semen analysis.

## Figures and Tables

**Table 1:** Pre-treatment and post-treatment semen parameters of patients who completed the study in the spermotrend- and placebo-groups.

Pre-Treatment			Post-Treatment	
Semen Parameters	Spermotrend-Group *n* = 35 *	Placebo-Group *n* = 35 *	Spermotrend-Group *n* = 35 *	Placebo-Group *n* = 35 *
	Median IQR			
Sperm total motility (a + b + c) (%)	52.0 42	57.0 35	60.0 44.9–62.6	58.0 43.9–63.8
Sperm rapid progressive motility (a) (%)	4.0 14	3.0 9	8.0 4.0–12.0	7.0 1.2–11.8
Sperm progressive motility (a + b) (%)	30.0 40.0	37.0 35.0	40.0 22.1–48.6	38.0 21.9–46.0
Sperm concentration (×10^6^/mL)	14.0 39	21.0 45	10.0 6.1–32.6	21.0 4.0–35.8
Sperm total number (×10^6^/mL)	32.9 99.8	36.0 106.1	24.0 14.1–104.7	40.3 18.7–67.8
Sperm morphology (normal forms) (%)	1.0 7	1.0 6	3.0 1.2–6.0	2.0 1.0–3.0
DFI (%)	27.0 20	35.0 29	23.0 18.5–32	30.0 17.7–41.7
	Mean SD			
Semen volume (mL)	3.4 2.0	2.9 1.6	3.3 2.6–3.9	2.9 2.3–3.5
8-OHdG (%)	10.7 4.5	11.3 5.1	9.1 7.8–10.4	8.7 7.6–9.9

Sperm total motility (a + b + c): progressive and non-progressive motility. Sperm motility (a): rapid progressive motility. Sperm motility (b): slow or sluggish progressive motility Sperm motility (a +b): progressive motility, sperm that move actively either in a straight line or large circles. Sperm motility (c): non-progressive motility, sperm that move but do not make forward progression. 8-OHdG: 8-hydroxy-2′-deoxyguanosine; DFI: DNA fragmentation index; SD: standard deviation; IQR: interquartile range. * In the placebo-group, due to an insufficient number of spermatozoa, 8-OH-dG analysis was not performed in one patient while DNA fragmentation analysis was not performed in two patients. Due to an insufficient number of spermatozoa, 8-OH-dG analysis was not performed in one patient in the placebo-group while DNA fragmentation analysis was not performed in two patients in the spermotrend-group.

**Table 2:** Changes of the pre-treatment and post-treatment semen parameters between the spermotrend- and placebo-groups.

	Spermotrend-Group *n* = 35 *		Placebo-Group *n* = 35 *	
Parameters	Median 95% CI	*p*-Value ** (Pre- vs. Post-Treatment)	Median 95% CI	*p*-Value ** (Pre- vs. Post-Treatment)
Sperm total motility (%)	0.0 −1.9 to +6.0	0.20	+4.0 −3.2 to +8.0	0.30
Sperm rapid progressive motility (a) (%)	+1.0 0.0 to +2.0	0.04	0.0 −0.2 to +3.0	0.06
Sperm progressive motility (a + b) (%)	+3.0 0.0 to +15.1	0.02	+1.0 −4.2 to +7.4	0.23
Sperm concentration (×10^6^/mL)	0 −2.8 to +4.4	0.64	+0.9 −0.9 to +3.0	0.53
	Mean 95% CI	*p*-value *** (Pre- vs. Post-treatment)	Mean 95% CI	*p*-value *** (Pre- vs. Post-treatment)
Sperm morphology (% nornal)	+0.5 −0.1 to +1.1	0.11	+0.4 −0.1 to +0.8	0.11
8-OHdG (%)	−1.7 −2.9 to −0.5	<0.01	−2.9 −4.6 to −1.2	<0.01
DFI (%)	−3.2 −5.8 to −0.5	0.02	−0.7 −6.2 to +0.4	0.08

Sperm total motility (a + b + c): progressive and non-progressive motility. Sperm motility (a): rapid progressive motility. Sperm motility (b): slow or sluggish progressive motility Sperm motility (a + b): progressive motility, sperm that move actively either in a straight line or large circles. 8-OHdG: 8-hydroxy-2-deoxy-guanosine; DFI: DNA fragmentation index. * Due to an insufficient number of spermatozoa, DNA fragmentation analysis was not performed in one patient in the placebo-group both before the initiation of treatment and after treatment completion. Moreover, for the same reason, DFI analysis was not possible in two patients prior to treatment initiation in the placebo-group and in one patient after treatment completion in the spermotrend-group. Overall, difference in DFI before and after the initiation of treatment could calculated in 32 patients in the placebo-group and in 34 patients in the spermotrend-group. * Due to an insufficient number of spermatozoa, 8-OH-dG analysis was not performed in one patient in the placebo-group before the initiation of treatment. Moreover, for the same reason, 8-OH-dG analysis was not possible in one patient after treatment completion in the placebo-group. Overall, difference in 8-OH-dG before and after the initiation of treatment could calculated in 33 patients in the placebo-group and in 35 patients in the spermotrend-group. ** Wilcoxon’s matched pairs rank-sum test. *** Paired *t*-test.

**Table 3:** Difference in the changes of pre- and post-treatment values between the spermotrend- and the placebo-groups.

Parameters	Difference in the Changes of Pre- and Post-Treatment Values Between the Spermotrend- and the Placebo-Groups *n* = 35 *	
	Mean 95% CI	*p*-value
Sperm progressive motility (a + b) (%)	−4.2 −11.1 to +2.6	0.22
Sperm morphology (% normal)	−0.1 −0.8 to +0.6	0.77
8-OHdG (%)	−1.2 −3.2 to +0.8	0.23
	Median ** 95% CI	*p*-value ***
Sperm total motility (a + b + c) (%)	0.0 −9.0 to +7.0	0.19
Sperm rapid progressive motility (a) (%)	0 −2.0 to +2.0	0.51
Sperm concentration (×10^6^/mL)	0.3 −6.6 to +6.0	0.68
DFI (%)	+1.0 −3.0 to +5.0	0.24

Sperm total motility (a + b + c): progressive and non-progressive motility. Sperm motility (a): rapid progressive motility. Sperm motility (b): slow or sluggish progressive motility Sperm motility (a + b): progressive motility, sperm that move actively either in a straight line or large circles. Sperm motility (c): non-progressive motility, sperm that move but do not make forward progression. 8-OHdG: 8-hydroxy-2-deoxy-guanosine; CI: confidence interval; DFI: DNA fragmentation index. * The differences between pre- and post-treatment values regarding sperm rapid progressive motility, sperm progressive motility, sperm total motility, sperm morphology, and sperm concentration could be compared between 35 patients in the spermotrend-group and 35 patients in the placebo-group who were completed the study. The difference between pre- and post-treatment values regarding DFI could be compared between 32 patients in the placebo-group and in 34 patients in the spermotrend-group. The difference between pre- and post-treatment values regarding 8-OHdG could be compared between 33 patients in the placebo-group and in 35 patients in the spermotrend-group. ** Hodges and Lehmann median difference. *** Quantile regression.

## Data Availability

The datasets generated during and/or analyzed during the current study are available from the corresponding author on reasonable request.

## Supplementary Materials

The following supporting information can be downloaded at: https://www.mdpi.com/article/10.3390/antiox14040488/s1. Supplementary Table S1. Characteristics of patients randomized in the spermotrend-group and in the placebo-group; Supplementary Table S2. Changes of pre-treatment and post- treatment semen parameters between the spermotrend and the placebo groups depending on the pre-treatment value of DFI; Supplementary Table S3. Difference in the changes of pre- and post-treatment values between the spermotrend and the placebo groups in patients depending on the pre-treatment value of DFI.
