# Supplementary material for: The Effect of Antioxidant Administration on Semen Quality in Men with Infertility: A Randomized Placebo-Controlled Clinical Trial
Source: Antioxidants (Basel). 2025 Apr 18;14(4):488. doi: 10.3390/antiox14040488 (PMC12024233; doi:10.3390/antiox14040488)
Supplement: Supplementary file 1 [file antioxidants-14-00488-s001.zip › antioxidants-3541111-supplementary.pdf]

Supplementary Table S1. Characteristics of patients randomized in the spermotrend-group and in the placebo-group

**Variable**	**Spermotrend-group n=40**	**Placebo-group n=40**
	Mean *SD*	
Age (years)	42.8 6.2	43.6 5.8
BMI (kg/m^2)	27.5 4.5	30.0 6.5
	Percentage (%) *n*	
Alcohol consumption (>7 units / week)	12.5% 5	30.7% 12
Smoking (current)	47.5% 19	37.5% 15
Exercise (>90 min / week)	45.0% 18	27.5% 11
Primary infertility	82.5% 33	82.5% 33

BMI: body mass index; CI: confidence interval; SD: standard deviation.

Supplementary Table S2. Changes of pre-treatment and post- treatment semen parameters between the spermotrend and the placebo groups depending on the pre-treatment value of DFI

DFI: DNA fragmentation index

Parameters	**Placebo** Median 95% CI **n=19**	**P-value**** ***(pre- vs. post-treatment)***	**Spermotrend** Median 95% CI **n=19**	**P-value**** ***(pre- vs. post-treatment)***	**Placebo** Median 95% CI **n=14**	**P-value**** ***(pre- vs. post-treatment)***	**Spermotrend** Median 95% CI **n=16**	**P-value* *** ***(pre- vs. post-treatment)***
Infertile men with pre-treatment DFI >25% **n=38***					***Infertile men with pre-treatment DFI <25%*** **n=30***			
**Sperm total motility (%)**	+4.0 -16.0 to +13.6	0.935	+2.0 -3.0 to +13.9	0.251	+3.0 -1.5 to +8.0	0.231	0.0 -3.0 to +5.5	0.622
**Sperm rapid progressive motility (a) (%)**	0.0 -0.3 to +2.3	0.159	+2.0 0.0 to +2.0	**0.022**	+1.5 -1.2 to +10	0.130	+0.5 -2.0 to +8.0	0.406
**Sperm progressive motility (a+b) (%)**	+1.0 -6.6 to +14.8	0.506	+5.0 -2.6 to +18.3	0.136	+5.5 -5.8 to +11.7	0.314	+2.5 -0.5 to +18.4	0.055
	Mean 95% CI	**P-value***** ***(pre- vs. post-treatment)***	Mean 95% CI	**P-value***** ***(pre- vs. post-treatment)***	Mean 95% CI	**P-value***** ***(pre- vs. post-treatment)***	Mean 95% CI	**P-value***** ***(pre- vs. post-treatment)***
**DFI (%)**	-4.6 -10.2 to +1.1	0.105	-5.0 -9.5 to -0.4	**0.032**	-0.5 -1.8 to +0.9	0.482	-0.9 -2.7 to 1.0	0.345
**8-OHdG (%)**	-2.5 -5.0 to -0.0	**0.049**	-1.2 -2.9 to +0.4	0.142	-3.4 -6.1 to -0.8	**0.014**	-2.2 -4.1 to -0.3	**0.024**

8-OHdG: 8-hydroxy-2-deoxy-guanosine

**Wilcoxon's matched pairs rank-sum test

***Paired t – test

*Due to insufficient number of spermatozoa, DNA fragmentation analysis was not performed in two patients prior to treatment initiation in the placebo-group.

Supplementary Table S3. Difference in the changes of pre- and post-treatment values between the spermotrend and the placebo groups in patients depending on the pre-treatment value of DFI

**Parameters**	**Difference in the changes of pre- and post-treatment values between the spermotrend and the placebo groups**<br><br>Mean<br>95% CI	**P-value***	**Difference in the changes of pre- and post-treatment values between the spermotrend and the placebo groups**<br><br>Mean<br>95% CI	**P-value***
	Infertile men with pre-treatment DFI >25%<br>**n=38**		***Infertile men with pre-treatment DFI <25%***<br>**n=30**	
**Sperm progressive motility (a+b) (%)**	-4.2<br>-14.8 to +6.2	0.417	-4.3<br>-14.3 to +5.6	0.378
**Sperm morphology (% normal)**	-0.2<br>-1.2 to +0.7	0.579	+0.1<br>-1.3 to +1.5	0.864
**8-OHdG (%)**	-1.3<br>-4.2 to +1.6	0.369	-1.2<br>-4.3 to +1.8	0.414
	Median**<br>95% CI	**P-value*****	Median**<br>95% CI	**P-value*****
**Sperm total motility (a+b+c) (%)**	-5.0<br>-22.0 to +9.0	0.804	+1.5<br>-5.0 to +7.0	0.619
**Sperm rapid progressive motility (a) (%)**	**0.0**<br>**-2.0 to +2.0**	**0.023**	+1.0<br>-3.0 to +7.0	0.590
**DFI (%)**	+0.0<br>-7.0 to +8.0	1.00	+1.0<br>-2.0 to +3.0	0.583
**Sperm concentration ($x10^6$/ml)**	+0.30<br>-5.1 to +5.0	0.879	-0.5<br>-32.0 to +18.0	0.961

DFI: DNA fragmentation index

8-OHdG: 8-hydroxy-2-deoxy-guanosine

*Paired t – test

** Hodges and Lehmann median difference.

***Quantile regression
